# Association between Barthel Index, Grip Strength, and Physical Activity Level at Admission and Prognosis in Community-Acquired Pneumonia: A Prospective Cohort Study

**DOI:** 10.3390/jcm11216326

**Published:** 2022-10-27

**Authors:** Camilla Koch Ryrsø, Maria Hein Hegelund, Arnold Matovu Dungu, Daniel Faurholt-Jepsen, Bente Klarlund Pedersen, Christian Ritz, Rikke Krogh-Madsen, Birgitte Lindegaard

**Affiliations:** 1 Department of Pulmonary and Infectious Diseases, Copenhagen University Hospital—North Zealand, 3400 Hillerød, Denmark; 2 Centre for Physical Activity Research, Copenhagen University Hospital—Rigshospitalet, 2100 Copenhagen, Denmark; 3 Department of Infectious Diseases, Copenhagen University Hospital—Rigshospitalet, 2100 Copenhagen, Denmark; 4 National Institute of Public Health, University of Southern Denmark, 1455 Copenhagen, Denmark; 5 Department of Infectious Diseases, Copenhagen University Hospital, Hvidovre, 2650 Copenhagen, Denmark; 6 Department of Clinical Medicine, University of Copenhagen, 2200 Copenhagen, Denmark

**Keywords:** Barthel index, community-acquired pneumonia, grip strength, intensive care unit, length of stay, mortality, readmission, physical activity

## Abstract

Background: Impaired functional status is a risk factor for hospitalization in patients with community-acquired pneumonia (CAP). The aim was to determine the influence of functional status and physical activity level on severe outcomes, including length of stay, admission to the intensive care unit (ICU), readmission, and mortality in patients with CAP. Methods: A prospective cohort study among patients hospitalized with CAP. Functional status was assessed with the Barthel index and grip strength, and physical activity level was assessed using the international physical activity questionnaire. Linear regression was used to assess the association with length of stay, and logistic regression was used to assess the risk of severe outcomes. Results: Among 355 patients admitted with CAP, 18% had a low Barthel index (<80), 45% had a low grip strength, and 75% had a low physical activity level. Low Barthel index was associated with increased risk of ICU admission (OR 3.6, 95% CI 1.2–10.9), longer length of stay (27.9%, 95% CI 2.3–59.7%), readmission within 30, 90, and 180 days (OR 2.1–2.4, *p* < 0.05), and mortality within 90 and 180 days (OR 4.2–5.0, *p* < 0.05). Low grip strength was associated with increased risk of 90 days readmission (OR 1.6, 95% CI 1.0–2.6, *p* < 0.05) and mortality within 30, 90, and 180 days (OR 2.6–3.2, *p* < 0.05). Low physical activity level was associated with increased risk of readmission within 90 and 180 days (OR 1.8–2.1, *p* < 0.05) and mortality within 30, 90, and 180 days (OR 3.3–5.5, *p* < 0.05). Conclusions: Impaired functional status and low physical activity level were associated with a longer length of stay and increased risk of ICU admission, readmission, and mortality in patients hospitalized with CAP. Routine assessment of functional status and physical activity level in clinical care could enable early identification of individuals with excess risk for a poor prognosis. Trial registration: ClinicalTrials.gov, NCT03795662.

## 1. Introduction

Community-acquired pneumonia (CAP) remains a leading cause of hospitalization and death from infectious diseases worldwide [1,2]. One in five patients is readmitted within 30 days [3], making readmissions a significant burden for patients and healthcare systems. Several assessment tools have been developed to stratify patients with CAP according to their mortality risk, such as the CURB-65 [4]. In recent years, increasing evidence has shown that functional status, measured with the Barthel index [5], a clinical tool for evaluating the ability to perform basic activities of daily living, adds essential information in predicting 1-year mortality in older hospitalized patients [6]. Further, the Barthel index appears to be a useful tool for evaluating patients at risk for a poor prognosis [7,8,9]. In addition, grip strength is commonly used to evaluate muscle function and serves as a marker for overall muscle strength and endurance [10,11]. In medical patients, lower grip strength measured at admission has been shown to be associated with prolonged length of stay and increased risk of in-hospital and 180-day mortality [12,13,14]. Finally, physical inactivity has been associated with an increased risk of readmission and mortality in hospitalized patients with an acute exacerbation of chronic obstructive pulmonary disease (COPD) [15,16]. The ability to identify individuals at risk of readmission and mortality after hospitalization for CAP would enable early initiation of target interventions and treatments for high-risk patients. Further, integrating assessment of functional status and physical activity level in the clinical practice might lead to improved prognosis among patients hospitalized with CAP. We hypothesized that patients with impaired functional status and low physical activity level would have an increased risk of severe outcomes after hospitalization with CAP. We aimed to examine the relationship between functional status (Barthel index and grip strength) and physical activity level and severe outcomes, including length of stay, admission to the intensive care unit (ICU), readmission (within 30, 90, and 180 days), and mortality (in-hospital and within 30, 90, and 180 days) in patients hospitalized with CAP. Secondly, we aimed to examine the relationship between changes in grip strength during admission and severe outcomes (readmission and mortality).

## 2. Methods

### 2.1. Study Design, Setting, and Patient Population

Patients were prospectively recruited at the Copenhagen University Hospital–North Zealand, Hillerød, Denmark, from January 2019 until July 2021. Inclusion criteria were age ≥18 years and suspected CAP defined as a new pulmonary infiltrate on chest X-ray or computed tomography scan and minimum one symptom consistent with CAP, e.g., fever (≥38.0 °C), hypothermia (<35.0 °C), cough, sputum production, pleuritic chest pain, dyspnoea, or focal chest signs on auscultation. Exclusion criteria were: no grip strength measurement at admission, length of stay < 24 h, and unable or unwilling to provide informed consent.

### 2.2. Data Collection

Information on demographic characteristics, comorbidities, symptoms, and prior medical history were collected from medical records. CURB-65 (confusion, urea, respiratory rate, blood pressure, and age ≥ 65 years) was used as a severity score, and patients were risk-stratified according to their CURB-65 score: mild (0–1), moderate (2), or severe (3–5) [4]. The combined burden of comorbidities was assessed using the Charlson Comorbidity Index [17] and categorized as 0, 1, or ≥2 comorbidities. Data on ICU admission, length of stay, and readmission (within 30, 90, and 180 days), including the cause of admission, were registered. Further, mortality (in-hospital and within 30, 90, and 180 days) was recorded. Data on readmission and mortality were collected up to 180 days after discharge.

Body weight was measured to the nearest 0.1 kg on an electronic scale (Seca, Hamburg, Germany), whereas height was self-reported. Body mass index was calculated as weight (kg)/height (m)^2^. Bioelectrical impedance analysis (BioScan touch i8 STD, Maltron International, Rayleigh, UK) was performed within 48 h of admission to determine fat-free mass as a proxy of total muscle mass. Hand-to-foot measurements of resistance and reactance were made using multi-frequency (5 kHz, 50 kHz, 100 kHz, 200 kHz). The patient was placed in a supine position with the extremities in a relaxed position, not touching the body. The current electrodes were placed on the foot, over the distal portion of the second metatarsal, and on the hand on the distal portion of the second metacarpal. The sensing electrodes were placed at the anterior ankle between the tibial and fibular malleoli and at the posterior wrist between the styloid processes of the radius and ulna. Fat-free mass was standardized to height, similar to body mass index:

Fat-free mass index = fat-free mass (kg)/height (m)^2^

Grip strength was measured within 48 h of admission with an electronic handheld dynamometer (Saehan Corporation, Gyeongsangnam-Do, South Korea) and based on the best attempt of three with the dominant hand. All measurements were performed seated with the shoulder and wrist in a neutral position and elbow flexed to 90°. A sex-specific cut-off for clinically relevant low grip strength suggested by Dodds and colleagues [11] was used: males <27 kg and females <16 kg.

The ability to perform basic activities of daily living was evaluated within 48 h of admission with the Barthel index [5], a score across ten domains, and weighted on a numerical scale, with the lowest score indicating total dependency and the highest score indicating complete independence. The score (0–100) was subcategorized into two standard diagnostic categories [18]: normal Barthel index ≥80 and low Barthel index <80.

Self-reported physical activity level was assessed at admission using the short form of the international physical activity questionnaire (IPAQ) [19]. According to the IPAQ guidelines [20], patients were categorized into three levels of physical activity (low < 600 metabolic equivalents of task (MET)-min/week, moderate ≥ 600 MET-min/week, or high ≥ 3000 MET-min/week).

### 2.3. Statistical Analysis

Data were described as counts (%) for categorical variables and either means (standard deviation (SD) or medians (interquartile range (IQR)) for continuous variables as appropriate. Model assumptions, including normality, were assessed using residuals and quantile-quantile plots. Due to the skewed distribution, the variable length of stay was log-transformed, and the proposed predictors, Barthel index, grip strength, and physical activity level, were assessed using a linear regression model. The regression coefficients were back-transformed to provide ratios. Binary logistic regression analyses were used to assess the risk of admission to the ICU, readmission, and mortality. To adjust for confounding, a stepwise selection process of covariates was performed. In the model, the following covariates were included: age, sex, fat-free mass index, COPD, and cancer. Results are reported as odds ratio (OR) with a corresponding 95% confidence interval (CI) and *p*-value. *p*-values were two-sided and significance levels were <0.05. Data were analyzed using IBM SPSS Statistics version 25.

## 3. Ethical Considerations

The study was approved by the scientific Ethics Committee at the Capital Region of Denmark (H-18024256), registered on ClinicalTrials.gov (NCT03795662), and conducted in accordance with the Declaration of Helsinki. Patients provided informed consent before enrolment.

## 4. Results

Among 705 patients screened for inclusion, 355 (50.4%) fulfilled the inclusion criteria and were included in this study. Grip strength was evaluated in 355 patients, Barthel index in 336 patients, and physical activity level in 326 patients upon admission. At discharge, grip strength was measured in 158 patients.

### 4.1. Demography, Comorbidities, and Clinical Parameters

Patient characteristics are summarized in Table 1. The mean age of the study population was 71 years, 66.8% had ≥2 comorbidities, and 51.1% had mild CAP (CURB-65 score of 0 or 1). During hospitalization, 16 patients (4.5%) were admitted to the ICU, while 50 patients (14.1%) refrained from admission to the ICU. In total, 18 patients (5.1%) died during admission, of which 11 refrained from admission to the ICU. The median length of stay was 6 days. Seventy-eight patients (23.1%) were readmitted within 30 days after discharge, while 31.7 and 42.0% of the patients were readmitted within 90 and 180 days after discharge, respectively. Pulmonary conditions (acute exacerbation of COPD, dyspnea, and acute respiratory failure) and pneumonia were the most common causes of readmission accounting for 27.5 and 21.8% of all readmissions, respectively (Supplementary Table S1). In total, 30 patients (8.5%) died within 30 days after discharge, while 48 (13.5%) and 63 patients (17.7%) died within 90 and 180 days after discharge, respectively (Table 1). For patients who died within 30 days after discharge, the most frequent causes of death were malignancies (50.0%), recurrence of CAP with respiratory failure (25.0%), chronic lung disease (16.7%), and cardiac disease (8.3%).

### 4.2. Barthel Index, Grip Strength, and Physical Activity

Overall, 18.2% had a Barthel index < 80, indicating a high dependency (Table 1). Low grip strength was seen in 44.5% and was more common for males than females (males 60.8% vs. females 26.6%, respectively). Overall, there was no change in grip strength during admission. In total, 43% of the patients had a mean loss of 2.7 kg (95% CI −3.4 to −2.1) in grip strength during admission, whereas 57% had a mean increase of 2.4 kg (95% CI 2.0 to 2.8). Based on IPAQ, 75% of the patients had a low physical activity level, while 17.8 and 7.1% had a moderate or high physical activity level, respectively.

### 4.3. Association between Barthel Index, Grip Strength, Physical Activity Level, and Prognosis

**Figure 1:**
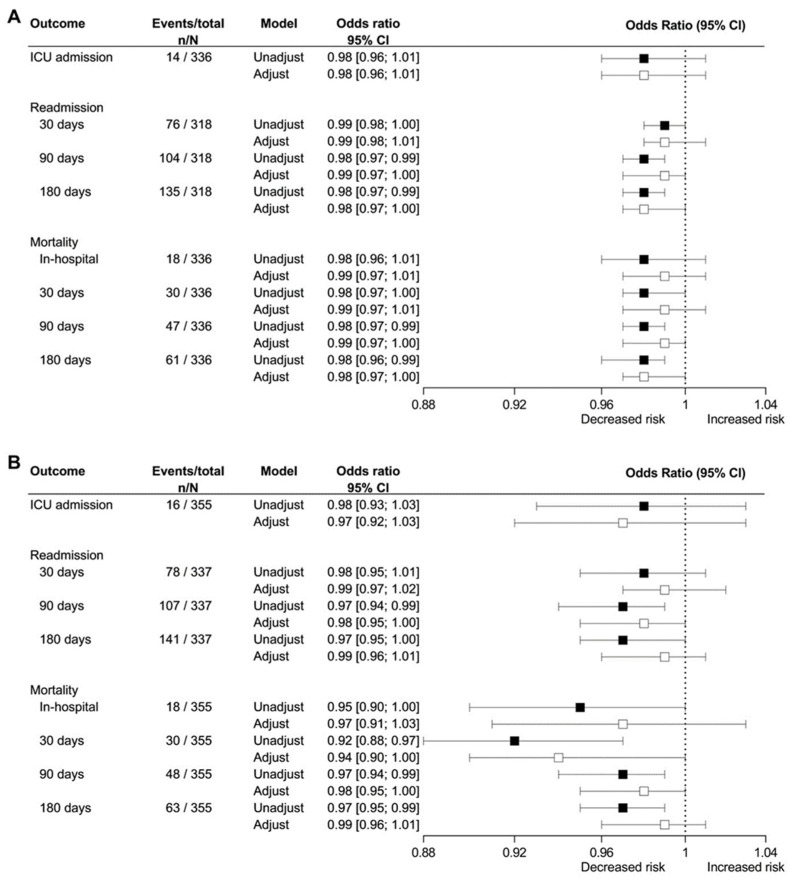
Association between Barthel index (**A**) and grip strength (**B**) and prognosis among 355 patients admitted with community-acquired pneumonia. The association between Barthel index and grip strength and risk for admission to the intensive care unit (ICU), readmission, and mortality were analyzed in an unadjusted logistic regression model (black) and a logistic regression model adjusted for age and sex (white). The patients who died in the hospital are not included in the analyses for readmission.

Length of stay was reduced by 0.70% (95% CI 0.03–1.29%, *p* < 0.05) for every 1-point increase in the Barthel Index. There was no association between grip strength or physical activity and length of stay. There was no association between Barthel index, grip strength, or physical activity and risk of ICU admission. The risk of 90 and 180 days readmission and mortality was reduced for every 1-point increase in the Barthel index (Figure 1A). Risk of 90 and 180 days readmission and 30, 90, and 180 days mortality were reduced for every 1 kg increase in grip strength (Figure 1B). However, the significant associations disappeared after adjusting for age and sex.

### 4.4. Association between Low Barthel Index, Low Grip Strength, Low Physical Activity Level, and Prognosis

**Figure 2:**
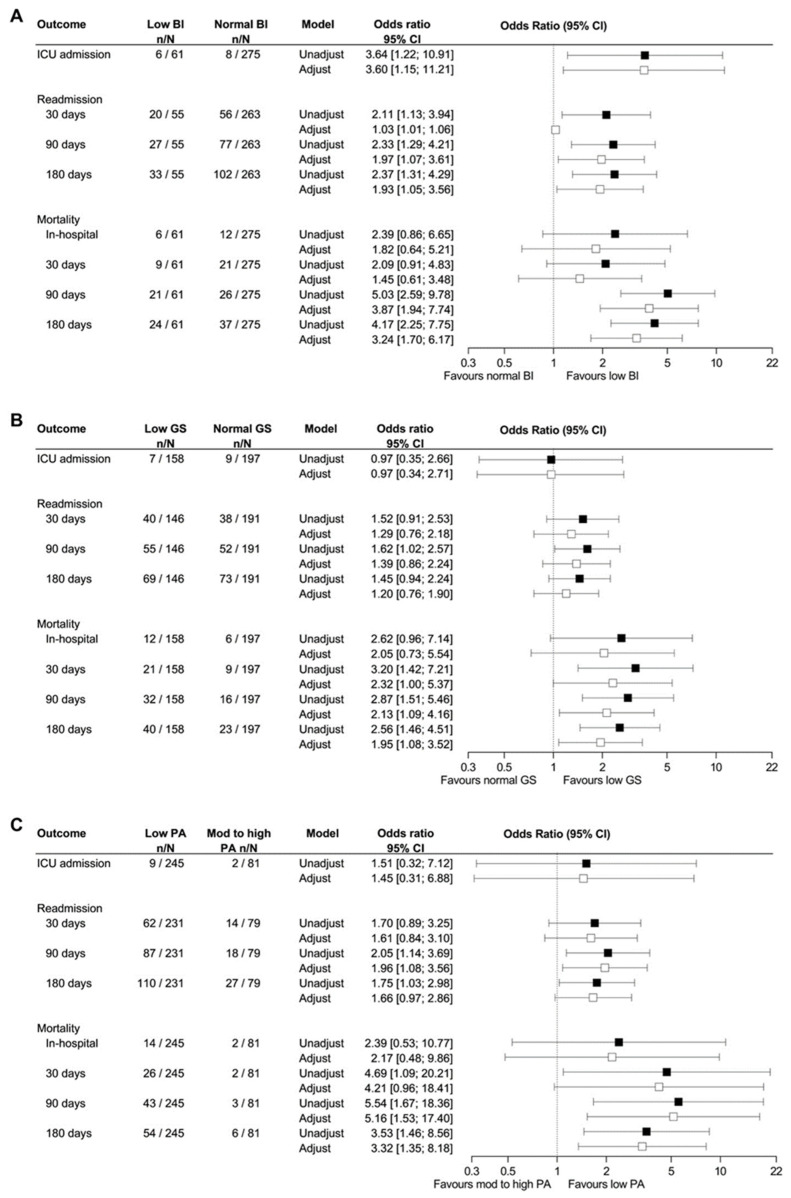
Association between low Barthel index (**A**), low grip strength (**B**), and low physical activity level (**C**) and prognosis among 355 patients admitted with community-acquired pneumonia. The association between low Barthel index, low grip strength, and low physical activity (PA) level and risk for admission to the intensive care unit (ICU), readmission, and mortality were analyzed in an unadjusted logistic regression model (black) and a logistic regression model adjusted for age (white). The patients who died in the hospital are not included in the analyses for readmission. Low grip strength: <27 kg for males and <16 kg for females. Low physical activity level: <600 MET-min/week vs. moderate (Mod) to high physical activity level: ≥600 MET-min/week. Low Barthel index <80.

Analysis was also performed with patients subcategorized into low (<80) or normal (≥80) Barthel index, low (females <16 kg and males <27 kg) or normal grip strength, and low (<600 MET-min/week) or moderate-to-high physical activity level (≥600 MET-min/week). Patients with low Barthel index had a longer length of stay (27.9%, 95% CI 2.3–59.7%, *p* < 0.05) and an increased risk of ICU admission (OR 3.6), readmission within 30, 90, and 180 days (OR 2.1–2.4), and mortality within 90 and 180 days after discharge (OR 4.2–5.0, Figure 2A). Patients with low grip strength had an increased risk of readmission within 90 days (OR 1.62) and mortality within 30, 90, and 180 days after discharge (OR 2.6–3.2, Figure 2B). Patients with low physical activity level had an increased risk of readmission within 90 and 180 days after discharge (OR 1.8–2.1) and mortality within 30, 90, and 180 days after discharge (OR 3.5–5.5, Figure 2C).

### 4.5. Association between Changes in Grip Strength during Admission and Prognosis

There was no association between changes in grip strength during admission and risk of readmission or mortality. However, patients who lost grip strength during admission seemed to be at a higher risk for mortality within 180 days after discharge (OR 2.2, 95% CI 0.95–5.14, *p* = 0.065).

## 5. Discussion

The present study clearly demonstrates that a low Barthel index is associated with an increased risk of ICU admission and a longer length of stay. Moreover, low Barthel index, grip strength, and physical activity level are associated with an increased risk of readmission and mortality up to 180 days after discharge. These findings demonstrate the importance of routine assessment of functional status and physical activity level as part of clinical care to identify patients at high risk of severe outcomes.

In patients with CAP, low functional status is commonly seen upon admission. In comparison, previous studies have reported that 27 to 40% of patients with CAP have a low Barthel index [7,21], a significantly higher number than the 18% seen in our study. However, in line with previous observations from patients with CAP [7,8,9,22], we showed that a low Barthel index was associated with a longer length of stay and increased risk of readmission and mortality. This is the first study showing an association between a low Barthel index and an increased risk of ICU admission in patients with CAP. In our study, only 4.5% of the patients were admitted to the ICU, a lower number than in previous observations from patients hospitalized with CAP [3,23]. However, in our study, 14.1% of the patients had a treatment limitation by refraining from admission to the ICU.

We found an increased risk of in-hospital mortality in patients with a low Barthel index, which is in line with previous observations from a Canadian study of 3043 patients with CAP [21], showing a 1.5-fold increased risk of in-hospital mortality in patients with walking difficulties compared to patients without difficulties. Compared to our findings, Murcia and colleagues [7] showed twice the risk as we did, with a 4-fold increased risk of 30-day mortality in patients with low Barthel index (≤80) after hospitalization with CAP. The slightly different findings could be explained by time differences in the assessment of the Barthel index, as we evaluated the Barthel index within 48 h of admission, whereas Murcia and colleagues [7] assessed it two weeks before admission.

In line with previous studies of medical patients [14,24,25], almost half of our patients had low grip strength. However, in contrast to the study by Mendes and colleagues [24] among hospitalized medical patients, we did not find any association between grip strength and length of stay. Differences in the percentage of patients with low grip strength might explain the different findings. In the study by Mendes and colleagues [24], 92.5% of the medical patients had low grip strength, whereas 44.5% of the patients had low grip strength in our study. However, more evidence is needed on the prognostic value of grip strength in patients with CAP. To our knowledge, this is the first study showing the association between low grip strength and increased risk of readmission and mortality in patients hospitalized with CAP. During hospitalization, no mean change in grip strength was found, even though 43% of the patients had a loss of grip strength. These findings are similar to previous observations from older medical patients [26]. However, these results diverged from our expectations as loss of muscle strength is common during hospitalization with CAP [27] due to the accelerated catabolic processes caused by acute inflammation and low physical activity level [28,29].

Despite the mounting evidence linking physical inactivity to an increased risk of mortality [30], this is the first study demonstrating an association between low physical activity level and increased risk of readmission and mortality after hospitalization with CAP. Previous studies have shown an increased risk of readmission and mortality in physically inactive patients after hospitalization with acute exacerbation of COPD [15,16]. However, a study of older medical patients has shown that exercise training during hospitalization reduces the risk of adverse events (e.g., prolonged length of stay, long-term care admission, and mortality) [31]. These findings suggest that increasing physical activity through exercise interventions could be a way to improve the prognosis of patients with CAP by reducing the risk of readmission and mortality.

Overall, 23.1% of the patients were readmitted within 30 days, a considerably higher number than the 16% previously reported in patients with CAP [3]. However, when we compared the cause of readmission within 30 days to the cause of readmission within 90 to 180 days after discharge, we found that 25.6% of the readmissions within 30 days were due to pneumonia, whereas 17.2% of the readmissions within 90 to 180 days after discharge were caused by pneumonia.

Several studies have consistently shown that routine assessment of functional status in hospitalized older adults adds crucial prognostic information beyond that provided by medical diagnosis or acute physiologic measures [6,32]. The reason for this might be that functional status reflects the severity and consequences of the combined burden of chronic diseases. In addition to functional status, frailty assessment has previously been used to predict adverse outcomes in patients with CAP [33]. Compared to functional status, which is the ability to perform activities of daily living and maintain health, frailty is characterized as diminished muscle strength, endurance, and reduced physiological function, which increases the vulnerability towards the development of increased dependency and mortality [34]. The variables we have used in our study to assess functional status and physical activity level are all included in the assessment tool for evaluating frailty [34], e.g., muscle weakness as measured with grip strength, functional ability measured with Barthel index, and physical activity level. However, in our study, we were specifically interested in exploring the association between reduced functional status and the risk of severe outcomes in patients with CAP. Several studies have shown that clinical markers of functional status, including grip strength, Barthel index, and physical activity level, are correlated to some extent, partly reflecting the same status of the patient [35,36]. However, to mirror what is relevant in clinical practice, we explored the association between the risk of severe outcomes with each marker of functional status individually.

The assessment of functional status in hospitalized patients extends well beyond its prognostic value as it also has important clinical implications for providing quality care during admission and after discharge. Indeed, functional status should be included in the risk stratification to identify patients at excess risk of severe outcomes and patients in need of physical rehabilitation. Future studies should focus on the impact of interventions aiming at improving functional status.

## 6. Study Strengths and Limitations

The main strength of our study was the prospective design, so real-time evaluation of functional status was possible. Further, as the study was a single-center study, geographic differences influencing the outcomes have been minimized.

Our study had some limitations. The first limitation of this study is the possibility of selection and inclusion bias, as informed consent had to be obtained within 24 h of admission, and grip strength had to be measured within 48 h of admission. We cannot rule out that patients’ refusal to participate in our study could be associated with the severity of their disease. Second, in patients hospitalized with CAP, prospective assessment of baseline functional status and physical activity level before the onset of illness is practically impossible. Even though we evaluated the functional status and physical activity level as close to admission as possible (within 48 h of admission), it is likely that the measured functional status and physical activity level upon admission had already been affected by CAP in the days leading up to admission.

## 7. Conclusions

The present study demonstrated that a low Barthel index, grip strength, and physical activity level are associated with a longer length of stay and a poor prognosis in patients hospitalized with CAP. Routine assessment of functional status and physical activity level, in addition to the clinical assessment, could improve the identification of patients at high risk of severe outcomes and lead to more targeted patient care with an increased focus on physical rehabilitation with the potential to improve the prognosis of vulnerable patients hospitalized with CAP.

## Figures and Tables

**Table 1:** Baseline characteristics of 355 patients hospitalized with community-acquired pneumonia.

	Study Population (*n* = 355)
Age, mean (SD), yrs.	71 (14)
Sex, male, *n* (%)	186 (52.4)
Nursing home resident, *n* (%)	15 (4.2)
Weight, median (IQR), kg	76.2 (64.8–88.4)
Body mass index, mean (SD), kg/m^2^	26.6 (6.2)
Fat-free mass index, mean (SD), kg/m^2^	17.9 (2.8)
Physical activity level based on the international physical activity questionnaire	
Low physical activity level, *n* (%)	245 (75.2)
Moderate physical activity level, *n* (%)	58 (17.8)
High physical activity level, *n* (%)	23 (7.1)
Grip strength, mean (SD), kg	23.9 (10.0)
Low grip strength	
Female (<16 kg), *n* (%)	45 (26.6)
Male (<27 kg), *n* (%)	113 (60.8)
Barthel index, median (IQR), score	100 (85–100)
≥80, score, *n* (%)	275 (81.8)
<80, score, *n* (%)	61 (18.2)
Number of comorbidities, *n* (%)	
0	49 (13.8)
1	69 (19.4)
≥2	237 (66.8)
Charlson Comorbidity Index, mean (SD)	4.7 (2.6)
CURB-65	
0–1, *n* (%), score	156 (51.1)
2, *n* (%), score	112 (36.7)
3–5, *n* (%), score	37 (12.1)
Admission to the intensive care unit, *n* (%)	16 (4.5)
Refraining from admission to the intensive care unit, *n* (%)	50 (14.1)
Length of stay, median (IQR), days	6 (3–9)
30 days readmission, *n* (%)	78 (23.1)
90 days readmission, *n* (%)	107 (31.7)
180 days readmission, *n* (%)	142 (42.0)
In-hospital mortality, *n* (%)	18 (5.1)
30 days mortality, *n* (%)	30 (8.5)
90 days mortality, *n* (%)	48 (13.5)
180 days mortality, *n* (%)	63 (17.7)

CURB-65: confusion, urea, respiratory rate, blood pressure, and age ≥65 years. Missing variables: weight (*n* = 20, 5.6%), body mass index (*n* = 22, 6.2%), fat-free mass index (*n* = 60, 16.9%), physical activity level (*n* = 29, 8.2%), Barthel index (*n* = 19, 5.4%), and CURB-65 (*n* = 50, 14.1%).

## Data Availability

Datasets used for the current study are not publicly available. However, relevant pseudonymized data can be accessed upon a reasonable request to the corresponding author.

## Supplementary Materials

The following supporting information can be downloaded at: https://www.mdpi.com/article/10.3390/jcm11216326/s1, STROBE Statement—a checklist of items that should be included in reports of observational studies; Table S1: Cause of readmission; Table S2: Etiology of the study population.

## Abbreviations

ADL: Activities of daily living; CAP: Community-acquired pneumonia; CCI: Charlson comorbidity index; CI: Confidence interval; COPD: Chronic obstructive pulmonary disease; CURB-65: confusion, urea, respiratory rate, blood pressure, age ≥65 years; ICU: Intensive care unit; IPAQ: International physical activity questionnaire; IQR: Interquartile range; MET: Metabolic equivalents of task; OR: Odds ratio; SD: standard deviation.
