# Supplementary material for: Association between Barthel Index, Grip Strength, and Physical Activity Level at Admission and Prognosis in Community-Acquired Pneumonia: A Prospective Cohort Study
Source: J Clin Med. 2022 Oct 27;11(21):6326. doi: 10.3390/jcm11216326 (PMC9653820; doi:10.3390/jcm11216326)
Supplement: Supplementary file 1 [file jcm-11-06326-s001.zip › Supplementary material_major revision.pdf]

Supplementary

Table S1. Cause of readmission

	Cause of 30-day readmission	Cause of 90 and 180-day readmission
Pulmonary, n (%)	22 (28.2)	17 (26.6)
Pneumonia, n (%)	20 (25.6)	11 (17.2)
Cardiovascular, n (%)	8 (10.3)	7 (10.9)
Malignant, n (%)	8 (10.3)	4 (6.3)
Infection, n (%)	2 (2.6)	9 (14.1)
Neurological, n (%)	7 (9.0)	3 (4.7)
Surgical, n (%)	1 (1.3)	1 (1.6)
Other, n (%)	10 (12.8)	12 (18.8)

Table S2. Etiology of study population

Pathogen, n (%)	Total (n = 355)
Bacteria	
Haemophilus influenzae	14 (3.9)
Streptococcus pneumoniae	12 (3.4)
Escherichia coli	12 (3.4)
Staphylococcus aureus	10 (2.8)
Legionella pneumophila	7 (2.0)
Klebsiella pneumonia	6 (1.7)
Pseudomonas aeruginosa	4 (1.1)
Mycoplasma pneumoniae	3 (0.8)
Moraxella catarrhalis	2 (0.6)
Virus	
Severe acute respiratory syndrome coronavirus 2	46 (13.0)
Influenza virus A	21 (5.9)
Human metapneumovirus	4 (1.1)
Respiratory syncytial virus	3 (0.8)
Parainfluenza virus	2 (0.6)
Other	53 (14.9)
